# Dietary Balance Index-07 and the Risk of Anemia in Middle Aged and Elderly People in Southwest China: A Cross Sectional Study

**DOI:** 10.3390/nu10020162

**Published:** 2018-01-31

**Authors:** Qiang Zhang, Guanghe Qin, Zhitao Liu, Zi Li, Juanjuan Li, Deepthi S. Varma, Qingqing Wan, Jiang Zhao, Xiangdong Min, Xingmeng Han, Min Liu

**Affiliations:** 1 Department of Nutrition and Food Hygiene, Yunnan Center for Disease Control and Prevention, Kunming 650022, China; zhangqiang_cs@aliyun.com (Q.Z.); ynguangheqin@yeah.net (G.Q.); zhitaoliu1976@163.com (Z.L.); yncdclizi@sina.com (Z.L.); juanjuanli402@163.com (J.L.); wanqingqing404@163.com (Q.W.); zhaojiang19761@sina.cn (J.Z.); xiangdongmin1965@tom.com (X.M.); 2 Department of Epidemiology, University of Florida, Gainesville, FL 32610, USA; dvarma@ufl.edu; 3 School of Public Health, Kunming Medical University, Kunming 650500, China; hanmengmeng1993@aliyun.com

**Keywords:** Dietary Balance Index, anemia, elderly population, Nutrition Survey, Chinese

## Abstract

A balanced diet is essential to achieve and maintain good health. In this study, we assessed diet quality of middle aged and elderly people based on Chinese Diet Balance Index-07 (DBI-07) and explored the associations between DBI-07 and anemia. Data analyzed for this study was from the 2010–2012 National Nutrition and Health Survey in Yunnan province, southwest China (*n* = 738, aged 50–77 years). Dietary recalls over there consecutive days were done in a face-to-face interview. The scores of DBI-07 for each component and three DBI-07 indicators ((Lower Bound Score (LBS), Higher Bound Score (HBS), Diet Quality Distance (DQD)) were calculated according to compliance with the Dietary Guidelines for Chinese residents. Hemoglobin (Hb) concentration was determined using the cyanmethemoglobin method. Univariate and multivariate linear regression models were used to explore the associations between DBI-07 indicators and anemia, as well as scores of DBI-07 components and Hb level. The sample included 336 men and 402 women. Inadequate intakes of vegetables, fruits, dairy, soybean, eggs, fish and excessive intakes of cereals, meat, cooking oil, salt were both common. 91.3% of the participants had moderate or high levels of inadequate food intake, while 37.7% had moderate or high levels of excessive food intake. The mean Hb was 14.2 ± 1.7 g/dL, with a prevalence of anemia of 13.0%. Subjects with high LBS and DQD were more likely to be anemic (all *p* < 0.05). After adjustment for potential confounders, there were positive correlations between Hb level and the intakes of vegetables and soybean (β_vegetables_ = 1.04, *p* < 0.01; β_soybean_ = 0.82, *p* = 0.04). In conclusion, dietary imbalance and anemia are common in middle aged and elderly population in southwest China and inadequate intakes of vegetables and soybean may increase the risk of anemia.

## 1. Introduction

Anemia remains a major public health concern globally [1]. Anemia is defined as a Hemoglobin (Hb) concentration below the established cut-off levels and thus a reduction in oxygen-carrying ability [2]. In the elderly population, anemia has been correlated with reduced quality of life, increased mortality and higher risk of cardiovascular diseases [3,4,5,6]. In the 2010–2012 China National Nutrition and Health Survey, the prevalence of anemia in older Chinese people was 12.6% [7]. As a result of increasing life expectancy and declining fertility rates, China is experiencing an ongoing aging process. It is estimated that one in four people will be 60 years and over by 2035 [8]. Therefore, preventing anemia and improving the health among middle aged and elderly in China is an urgent need of the hour.

Poor nutrition has been identified as the main cause of anemia [9]. People with iron deficiency anemia frequently show micronutrient deficiencies (especially iron, folate, zinc, Vitamin A and D) [10,11]. Following rapid economic and social development, animal food consumption in Chinese population increased from 117 g/day in 1992 to 162.4 g/day in 2010–2012 and correspondingly the prevalence of anemia actually declined from 20.7% to 9.7% [7,12,13]. Despite this increased animal food consumption, anemia remains high in certain economically well-developed regions in China [14]. Shi et al. found that inadequate riboflavin intake was associated with increased risk of anemia in Chinese adults [15]. Ha et al. reported that high consumption of iron can cause copper-deficiency anemia in rats [16]. These findings highlight the complex effects of micronutrients on anemia that require further investigation. Moreover, some important covariates like total energy intake and physical activity are seldom considered in studies of diet and anemia [17,18]. Traditional nutritional analyses which merely focus on individual nutrients or foods, has limitations to interpret the associations between diet intake and anemia. For example, in a study of Chinese elderly population, red meat consumption was identified as a risk factor for anemia, which should have been a protective factor for anemia [19].

Recent nutritional studies have shifted towards examining diet-diseases associations using overall diet rather than focusing on individual foods or nutrients [20,21]. Based on current nutrition knowledge, several diet quality indices have been developed to evaluate the healthfulness of individual diets. For instance, the Healthy Eating Index (HEI) aims to assess dietary pattern and diet changes in the US population [22]. The Mediterranean Diet Score (MDS) has been used to estimate population adherence to the traditional Mediterranean Diet which shows a protective effect on chronic diseases [23]. The Chinese Dietary Balance Index-07 (DBI-07) was designed to evaluate under and over nutrition which have been identified as important risks for the rise of Non-Communicable Diseases (NCDs) in the Chinese population [24]. The validity of DBI-07 has been widely verified in assessing diet quality among subgroups in China [25]. Recent studies have explored the associations between DBI-07 and health indicators like metabolic syndrome and bone mineral density [26,27].

In this study, we evaluated diet quality using DBI-07 among middle aged and elderly people in southwest China, which is a less developed region with a high prevalence of anemia [13]. In addition, we examined the associations between scores of DBI-07 and anemia, as well as Hb level.

## 2. Materials and Methods

### 2.1. Study Design

Data used in this study were obtained from the 2010–2012 China National Nutrition and Health Survey in Yunnan Province, southwest China. Participants were recruited from two urban districts and four rural counties using a stratified cluster sampling method. From each of the six counties/districts, which are at different levels of economic development, three townships were randomly selected. Next, two villages were randomly selected from each township and 30 households were randomly selected from each village. All members aged 2 years or over in the households were invited to participate, with a response rate of 96.0%. Altogether, 778 participants aged 50 years or over completed the survey. 40 participants were excluded since they reported to be on a prescribed diet (14 subjects) or having serious illnesses (26 subjects). The final sample analyzed for this paper was 738 participants.

The ethical approval was obtained from the Institutional Review Board at China Centers for Disease Control and Prevention (2013–2018). Before administrating the survey, signed informed consent was obtained from all the participants.

### 2.2. Dietary Data Collection

Diet was assessed with 24 h recalls and a household food inventory over three consecutive days (including two weekdays and one weekend day). Every household member aged 2 or over was asked to report all food consumed, whether at home or away from home. With food picture and model aids, trained interviewers recorded the types and the amounts of food consumed at each meal via face to face interview. Condiments (such as salt, sauce and etc.) and cooking oils consumption at the household level were measured by the changes in a household food inventory over the period. Individual consumptions of cooking oil and salt were computed according to per capita consumption in each household and then adjusted by individual energy intake.

Data of alcohol consumption was derived from the lifestyle survey. Participants were inquired the drinking frequency and quantity of alcoholic beverages (such as high alcohol liquor, low alcohol liquor, wine, beer and etc.) in the past 12 months. Alcohol in each alcoholic beverage was converted following DBI-07 criteria [24] and then summed to calculate average alcohol consumption per day. After carefully reviewed, dietary data was summed by food group classifications correspond with the Chinese Food Composition Table for further analysis [28].

### 2.3. Dietary Balance Index-07

DBI-07 has been devised to assess diet quality in the Chinese population. DBI-07 comprises seven components from ‘the Dietary Guidelines for Chinese residents,’ including: (1) cereals; (2) vegetables and fruits; (3) milk and dairy products, soybean and soybean products; (4) Animal food; (5) condiments and alcoholic beverage; (6) Diet variety; (7) drinking water. A score of 0 for each DBI-07 component indicates meeting the recommended intake amounts. Positive scores (range 0–12) were used to assess excessive intake levels of alcoholic beverages and condiments, which the guidelines recommended consuming in a “reduced” or “limited” amount. Similarly, negative scores (range −12–0) were used to assess inadequate intake levels of vegetables and fruits, dairy and soybeans, dietary diversity and drinking water, which the guidelines recommended that people should consumed in a “sufficient” or “plenty” amount. Moreover, both positive and negative scores were used to evaluate the intake levels of cereals (−12–12) and animal food (−12–8), which the guidelines recommend consuming in an “appropriate” amount. In considering the difference of nutrient requirements in energy consumption, the scoring of cereals, vegetables, fruits, dairy, soybean, animal food and cooking oil is based on seven energy intake levels. The intakes of 12 identified food subgroups are used to assess the diet variety component of DBI-07. These food subgroups were (1) rice and products; (2) wheat and products; (3) corn, coarse grains and products, starchy roots and products; (4) dark-colored vegetables; (5) light-colored vegetables; (6) fruits; (7) soybean and products; (8) milk and dairy products; (9) red meat and products; (10) poultry and game; (11) egg; (12) fish and shellfish. A score of 0 is assigned to a food subgroup if the intake reached or exceeded the lowest recommended intake. Else, a score of −1 is assigned. The lowest recommended intake is 5 g for soybean and products and 25 g for other 11 food subgroups. The score of diet variety component ranges from −12 to 0 [24,29,30]. Scoring details of DBI-07 can be found in Table S1.

Based on the scores for each DBI-07 component, there indicators of diet quality are calculated. Higher Bound Score (HBS), the indicator for excessive food intake, is calculated by adding all the positive scores. Lower Bound Score (LBS), the indicator for inadequate food intake, is computed by adding all the negative scores. Diet Quality Distance (DQD), the indicator of imbalanced food intake, is calculated by the absolute values of both positive and negative scores. Data on water consumption was not available in the survey and therefore, the component of ‘drinking water’ was not assessed in this study. Therefore, the lower and upper bound of HBS, LBS and DQD were: −72 to 44, 0 to 32, 0 to 60 and 0 to 72, respectively. Each indicator is further divided into five levels to reflect diet quality for simplicity. They are “no problem” (a score of 0), “almost no problem” (less than 20% of the total score), “low level” (between 20% and 40% of the total score), “moderate level” (between 40% and 60% of the total score) and “high level” (greater than 60% of the total score) [24,29,30]. Contribution rates were calculated by dividing total scores of each DBI-07 component respectively by total scores of LBS, HBS and DQD, to measure the effect of each food subgroup intakes on diet quality.

### 2.4. Blood Sample, Hb Measurement and Criteria of Anemia

Six milliliter venous blood was collected from each participant using EDTA-containing vacuum tubes. Hemoglobin concentration was determined by the cyanmethemoglobin method. All local laboratory technicians have participated in a national training and passed a qualifying examination. In field examination, one quality control sample was tested in every 30 samples to ensure quality control. The residential elevation above sea level (altitude) for each township was obtained from Google Earth Software (Google Inc., Mountain View, CA, USA). Hb concentrations for the diagnosis of anemia was <13 g/L for men and <12 g/dL for women, all Hb values were adjusted for altitude and smoking behaviors (derived from the lifestyle survey) [31].

### 2.5. Other Variables

Height and weight were measured without shoes and heavy clothes, by trained local health workers. Body Mass Index (BMI) was computed as measured weight (in kilogram) divided by measured height in meter squared. The BMI cut-off points for Chinese adults were used to determine underweight (BMI < 18.5 kg/m^2^), normal weight (BMI 18.5–23.9 kg/m^2^) and overweight and obesity (BMI ≥ 24.0 kg/m^2^) [32]. Age groups were divided into two categories (50–59 and 60–77 years). Place of residence was determined by the sampling location (rural/urban). Education level was allocated into two categories (middle school or higher/primary school or lower). Household income in the last year was classified into two levels (0–10,000 RMB/person and 10,000–20,000 RMB/person).

### 2.6. Statistical Analysis

Numeric variables which normally distributed (e.g., the DBI-07 score for each component and the three DBI-07 indicators) were given as mean ± standard deviation (SD). Categorical variables (e.g., sex, age groups and the prevalence of anemia) were given as a percentage (%). Univariate and multivariate linear regression models were used to examine the associations between DBI-07 indicators and socio-demographic predictors, as well as anemia. Multivariate linear regression models were used to evaluate the associations between DBI-07 scores for each component and Hb level. All analyses were performed with SAS 9.4 (SAS Institute, Cary, NC, USA) and statistical significance was set at *p* < 0.05.

## 3. Results

Table 1 shows the socio-demographic characteristics of the participants. There were 738 participants in this study (336 men and 402 women). The mean age was 61.6 ± 8.3 years. 58.1% of the participants were from rural areas and nearly 70% had primary education or no formal education. Approximately 10% of the participants were underweight (BMI < 18.5 kg/m^2^). The overall prevalence of anemia was 13.0% (12.2% in men and 13.7% in women).

Table 2 shows the scores for the DBI-07 components and the percentage of participants with each score. Excessive cereals, meat, oil and salt intakes were common, with 84.9%, 47.4%, 57.2% and 58.0% of the participants being given a positive score. In contrast, inadequate intakes of vegetables and fruits, dairy and soybean, fish and eggs were also common, with nearly 80–99% of the participants being given a negative score. Diet variety was very poor in the participants, with all participants in the negative range. Most participants drank alcoholic beverages appropriately.

Table 3 presents the distribution of DBI indicators among the participants. The distribution of LBS indicates that 24.9% of the participants had high level of inadequate food intake. The distribution of LBS indicates that 4.5% of the participants had high level of excessive food intake. According to the distribution of DQD, the indicator of imbalanced food intake, more than half (54.7%) of people had high level of imbalanced food intake. The mean scores for LBS, HBS and DQD were 32.5, 11.5 and 44.1, respectively.

The contribution rates of DBI-07 components to LBS, HBS and DQD were presented in Table 4. The top three contributors to LBS were diet variety, dairy and fruits. The top contributor to HBS was cereals. The top three contributors to DQD were diet variety, cereals and dairy.

Table 5 shows the DBI-07 indicators by predictors. People living in rural areas, of lesser educational qualifications, of lower income, being underweight and having anemia had a higher LBS score (*p* < 0.05). People living in rural areas, males and aged 50–59 years had a higher HBS score (*p* < 0.05). DQD scores were higher among people living in rural areas, of lesser education, of lower income, being underweight and anemic (*p* < 0.05).

Table 6 presents the multivariable linear regression models for Hb levels by DBI-07 components. After adjustment for age, sex, BMI, education, income and place of residence, Hb level was positively correlated with the DBI-07 components of vegetables and soybean intakes (β_vegetables_ = 1.04, *p* < 0.01; β_soybean_ = 0.82, *p* = 0.04).

## 4. Discussion

The findings of this cross-sectional study can be summarized as follows. Firstly, diet imbalance is common among middle aged and elderly people in southwest China, especially for those living in rural, of lower education, of lower income. Secondly, people with anemia were more likely to be inadequate intake of food (LBS) and imbalanced intake of food (DQD). Finally, Hb level was positively correlated with the DBI-07 components of vegetables and soybean intakes.

Over the past 20 years, China has experienced an ongoing nutrition transition, which mainly characterized by decreased cereals intake and increased oil and animal food intakes [33]. In contrast to a previous study from the developed areas of China, middle aged and elderly people in southwest China has a higher percentage of excessive cereals intake (84.9% vs. 50%) [30]. Meanwhile, coarse grains consumptions have dropped quickly in Chinese population, which greatly reduced the intakes of some important micronutrients like dietary fibers [34]. Excessive cereals intake and decreased coarse grains consumption in their staple food is currently a common problem among Chinese people. Moreover, nearly half of participants also reported moderate or high levels of excessive meat, oil and salt intakes. Such high-fat, high energy-density dietary pattern has been identified as a main cause of NCDs in China [35].

Our results also show that inadequate intakes of vegetables, fruits, dairy, soybean, fish and egg were common in middle aged and elderly population. Over the past decade, consumptions of vegetables and fruits have remained almost unchanged in China, which only meets half of the recommendations [36]. In particular, dark-colored vegetables account for merely a third. The National representative study shows that the consumptions of all animal foods except meat are far below the recommendations [7]. For example, although the price of eggs has been constant, the consumption of eggs only varied from 23.7 g in 2002 to 24.3 g in 2012 [33]. In contrast, the consumption of meat had exceeded the recommendations in 2002 (79 g/day) and continued to increase to 89.7 g/day in 2012, although the price have doubled in the ten years [33]. Those findings suggest that in addition to economic factors, other factors like diet habits and nutrition knowledge are equally important for following a healthy diet. Moreover, the consumption of dairy products in Chinese people was 24.7 g/day on average, only 10% of the recommendations [36]. Supply shortage of dairy products and high prevalence of lactose intolerance are identified as two important factors for extremely low dairy consumptions among Chinese adults [37,38]. Therefore, boosting supply and providing other types of dairy products (e.g., yogurt and cheese) may increase dairy consumption.

Previous studies have shown that diet quality is generally determined by socio-demographic factors [39,40]. In this study, we found that high education and high income were protective factors for inadequate food intake, while female gender and older age (>60 years) are protective factors for excessive food intake. Our findings that urban residency is a protective factor for both inadequate food intake and excessive food intake are consistent with a previous study in Shanghai [30]. One possible explanation would be that food supply and accessibility, income and nutrition knowledge are better for rural residents. These findings underscore the necessity of nutrition education in rural areas of China. Moreover, we found that LBS scores and DQD scores were significantly higher in those who were underweight. This finding indicates that diet imbalance (especially inadequate intake) remains one of the most important nutritional problems among middle aged and elderly population in southwest China. Our finding is also consistent with a national representative study which examined the sensitivity of DBI-07 scores on BMI [29].

Adhering to evidence-based dietary guidelines is promising for elderly people to achieve and maintain good health. However, the associations between dietary balance and anemia have rarely been investigated, especially in elderly population. A previous study conducted among elderly women in southwest China found that women with anemia usually had higher LBS [41]. In this study, there were positive associations between the intakes of vegetables and soybean and Hb levels. Coincidently, a healthy dietary pattern (which was negatively associated with anemia) identified by a posteriori method was similarly characterized by high vegetable and soybean intakes [14]. For older Chinese population, vegetables are the main sources of vitamin C (account for 80–90%), which can improve the absorption of iron in diet [42]. A previous study observed that iron absorption was significantly improved by consuming 25 mg Vitamin C twice daily in Mexico women [43]. However, according to the data from the China Health and Nutrition Survey (CHNS), the intake of Vitamin C in rural elderly population had decreased by 12 mg from 1991 to 2009 due to reduced vegetables consumption and only 33.0% of the people met the recommendation of 100 mg/day [21]. A number of studies on diet and anemia indicated that soy food intake was a protective factor in older Chinese population [19,44]. One possible reason is that soybean was rich in iron and which is highly absorbed [45]. Another possible mechanism is that water-soluble soybean fiber can promote iron absorption and prevent anemia [46]. Moreover, soybean is a good and inexpensive source of protein. Because anemia in the older Chinese population is usually associated with poor protein nutrition and low income, preventing anemia by increasing the intakes of soybean and products might be a promising strategy [44,47]. The strength of our study is that the scores of DBI-07 is based on different levels of energy consumption like HEI, thus the potential confounding effect of total energy intake are appropriately controlled [48].

We acknowledge that this study has potential limitations. First, the cross-sectional nature of the study restricts casual inference between dietary intakes and anemia. Second, dietary recalls over three consecutive days are inadequate to assess dietary intakes over a long term. As Zhang et al. reported, seasonal variations in dietary intakes are significant, even in economically well-developed areas of China [30]. Third, the DBI-07 has been developed for general Chinese population, thus several extra recommendations for elderly population in the guidelines are not included in the current DBI-07. Forth, due to data limitation, other potential and non-nutritional causes of anemia (e.g., chronic kidney disease, H pylori and parasite infections), have not involved and controlled in this study. Last but not least, this analysis is only based on a sample of 768 adults aged 50–77 years from southwest China, which limits the generalization of the findings.

## 5. Conclusions

In conclusion, imbalanced diet intake and anemia are common in middle aged and elderly population in southwest China and inadequate intake of vegetables and soybeans may increase the risk of anemia. Nevertheless, prospective studies are needed to explore possible mechanisms.

## Figures and Tables

**Table 1:** Socio-demographic characteristics of the participants.

	Men	Women	Overall
N	336	402	738
Mean age in years (X ± SD)	62.2 ± 8.6	61.1 ± 8.1	61.6 ± 8.3
Age groups (%)			
50–59	48.2	53.7	45.5
60–77	51.8	46.3	54.5
Place of residence (%)			
Urban	41.1	42.5	41.9
Rural	58.9	57.5	58.1
Education level (%)			
Primary school or lower	62.8	74.9	69.4
Middle school or higher	37.2	25.1	30.6
Income in Yuan/person (%)			
0–10,000	64.3	68.4	66.5
10,000–20,000	35.7	31.6	33.5
BMI, kg/m^2^ (%)			
15.7–18.4	11.6	9.7	10.6
18.5–23.9	51.2	41.5	45.9
24.0–33.6	37.2	48.8	43.5
Anemia (%)	12.2	13.7	13.0

**Table 2:** Scores for the DBI-07 components and the percentage of the participants with each score.

Score	Cereals	Vegetables and Fruits		Dairy and Soybean		Animal Food			Oil and Condiments			Diet Variety
		Vegetables	Fruits	Dairy	Soybean	Meat	Fish	Egg	Oil	Salt	Alcohol	
(−12)–(−11)	0.1											0.7
(−10)–(−9)	0.4											12.8
(−8)–(−7)	0.4											49.1
(−6)–(−5)	0.3	0.8	68.7	93.4	59.9							30.5
(−4)–(−3)	2.3	42.0	21.1	4.2	31.0	12.2	91.7	58.5				6.2
(−2)–(−1)	3.7	37.9	6.8	2.3	5.6	9.4	3.7	18.6				0.7
0	8.0	19.2	3.4	0.5	3.5	31.0	4.6	14.6	45.8	42.0	96.8	0
1–2	12.2					20.3			21.0	37.5	2.3	
3–4	15.2					27.1			33.2	20.5	0.9	
5–6	20.8											
7–8	17.5											
9–10	7.9											
11–12	6.3											
Mean score	6.2	−2.5	−5.1	−5.8	−4.9	0.8	−3.7	−2.5	1.7	1.6	0.1	−6.9

**Table 3:** Distribution of DBI-07 indicators among the participants.

	Indicator	Range	Mean (SD)	Distribution of Diet Quality (%)				
				No Problem	Almost No Problem	Low Level	Moderate Level	High Level
Inadequate intake	LBS	0–60	32.5 (5.8)	0	0	8.7	66.4	24.9
Excessive intake	HBS	0–32	11.5 (4.6)	0.4	17.8	44.2	33.2	4.5
Overall imbalance	DQD	0–72	44.1 (7.1)	0	0	2.9	42.4	54.7

**Table 4:** Contribution rates of DBI-07 components to LBS, HBS and DQD.

LBS (%)		HBS (%)		DQD (%)	
Diet variety	21.1	Cereals	56.9	Diet variety	15.8
Dairy	17.7	Meat	13.2	Cereals	15.5
Fruits	15.7	Oil	15.5	Dairy	13.2
Soybean	15.2	Salt	13.9	Fruit	11.7
Fish	11.4	Alcohol	0.6	Soybean	11.3
Egg	8.3			Fish	8.5
Vegetables	7.6			Egg	5.7
Meat	2.1			Vegetables	5.7
Cereals	0.8			Meat	5.0
				Oil	4.0
				Salt	3.6
				Alcohol	0.1

Note: DBI-07 components are presented in descending order according to their contribution rates to LBS, HBS and DQD.

**Table 5:** DBI-07 indicators by predictors.

	LBS	*p*	HBS	*p*	DQD	*p*
	Mean (SD)		Mean (SD)		Mean (SD)	
Sex						
Men	32.4 (5.9)	0.59	12.1 (4.6)	<0.01	44.5 (7.2)	0.17
Women	32.6 (5.7)		11.1 (4.6)		43.7 (7.1)	
Age groups						
50–59	32.6 (5.0)	0.70	12.0 (4.8)	<0.01	44.6 (6.6)	0.06
60–77	32.5 (6.5)		11.1 (4.4)		43.8 (7.6)	
Place of residence						
Urban	29.8 (6.4)	<0.01	10.6 (5.4)	<0.01	40.5 (7.0)	<0.01
Rural	34.5 (4.3)		12.2 (3.8)		46.7 (6.0)	
Education level						
Primary or lower	33.8 (4.9)	<0.01	11.7 (4.4)	0.08	45.5 (6.4)	<0.01
Middle or higher	29.6 (6.6)		11.1 (4.9)		40.7 (7.5)	
Income in Yuan/person						
0–10,000	33.6 (5.0)	<0.01	11.4 (4.5)	0.37	45.1 (6.6)	<0.01
10,000–20,000	30.3 (6.5)		11.7 (4.8)		42.1 (7.8)	
BMI						
15.7–18.5	34.1 (3.9)	<0.01	10.5 (4.7)	0.09	44.6 (5.1)	<0.01
18.5–23.9	32.2 (5.9)		11.7 (4.4)		43.9 (7.3)	
24.0–33.6	31.5 (5.8)		11.6 (4.7)		43.0 (7.3)	
Anemia						
Yes	33.7 (4.6)	0.03	12.3 (4.3)	0.10	46.0 (6.6)	<0.01
No	32.3 (5.9)		11.4 (4.6)		43.8 (7.2)	

Note: values in bold are statistically significant at the 0.05 level.

**Table 6:** Multivariable linear regression models for Hb levels by DBI-07 components.

		β	*p*
Cereals		−0.12	0.36
Vegetables and fruits	Vegetables	1.04	0.01
	Fruits	0.15	0.74
Dairy and soybean	Dairy	0.98	0.19
	Soybean	0.82	0.04
Animal food	Meat	−0.44	0.10
	Fish	0.55	0.42
	Egg	0.12	0.72
Condiments and alcohol	Oil	−0.26	0.48
	Salt	−0.25	0.57
	Alcohol	−0.42	0.78
Diet variety		0.16	0.79

Note: adjustment for age, sex, BMI, education, income and place of residence and values in bold are statistically significant at the 0.05 level.

## Supplementary Materials

The following are available online at http://www.mdpi.com/2072-6643/10/2/162/s1. Table S1: components of the Chinese dietary balance index-revised. Table S2: The Dietary Guidelines for Chinese residence and the corresponding components for DBI-07. Table S3: elements of diet variety score.

## References

[1] Kassebaum N.J., Jasrasaria R., Naghavi M., Wulf S.K., Johns N., Lozano R., Regan M., Weatherall D., Chou D.P., Eisele T.P. (2014). A systematic analysis of global anemia burden from 1990 to 2010. Blood.

[2] World Health Organization (2001). Iron Deficiency Anaemia: Assessment, Prevention and Control: A Guide for Programme Managers. http://www.who.int/nutrition/publications/micronutrients/anaemia_iron_deficiency/WHO_NHD_01.3/en/.

[3] Lucca U., Tettamanti M., Mosconi P., Apolone G., Gandini F., Nobili A., Tallone M.V., Detoma P., Giacomin A., Clerico M. (2008). Association of mild anemia with cognitive, functional, mood and quality of life outcomes in the elderly: The “health and anemia” study. PLoS ONE.

[4] Hong C.T., Huang Y.H., Liu H.Y., Chiou H.Y., Chan L., Chien L.N. (2016). Newly diagnosed anemia increases risk of parkinson’s disease: A population-based cohort study. Sci. Rep..

[5] Chang Y.L., Hung S.H., Ling W., Lin H.C., Li H.C., Chung S.D. (2013). Association between ischemic stroke and iron-deficiency anemia: A population-based study. PLoS ONE.

[6] Lyu Y., Yin Z., Luo J., Shi X., Zeng Y. (2015). Association between anemia and 3-year all-cause mortality among oldest old people in longevity areas in China. Chin. J. Epidemiol..

[7] Chang J.L., Wang Y. (2016). Reports of China Nutrition and Health Survey (2010–2013).

[8] World Health Organization (2011). Country Health Information Profiles-China. http://www.wpro.who.int/countries/chn/5CHNpro2011_finaldraft.pdf?ua=1.

[9] Haider B.A., Yakoob M.Y., Bhutta Z.A. (2011). Effect of multiple micronutrient supplementation during pregnancy on maternal and birth outcomes. BMC Public Health.

[10] Sazawal S., Dhingra U., Dhingra P., Hiremath G., Sarkar A., Dutta A., Menon V.P., Black R.E. (2010). Micronutrient fortified milk improves iron status, anemia and growth among children 1–4 years: A double masked, randomized, controlled trial. PLoS ONE.

[11] Bird J.K., Murphy R.A., Ciappio E.D., McBurney M.I. (2017). Risk of deficiency in multiple concurrent micronutrients in children and adults in the United States. Nutrients.

[12] Zhai F.Y., He Y.N., Ma G.S., Li Y.P., Wang Z.H., Hu Y.S., Zhao L.Y., Cui Z.H., Li Y., Yang X.G. (2005). Study on the current status and trend of food consumption among Chinese population. Chin. J. Epidemiol..

[13] Yang X.G., Zhai F.Y. (2006). Chinese Nutrtion and Health Survey: Report Three.

[14] Shi Z., Hu X., Yuan B., Pan X., Dai Y., Holmboe-Ottesen G. (2006). Association between dietary patterns and anaemia in adults from jiangsu province in eastern China. Br. J. Nutr..

[15] Shi Z., Zhen S., Wittert G.A., Yuan B., Zuo H., Taylor A.W. (2014). Inadequate riboflavin intake and anemia risk in a chinese population: Five-year follow up of the Jiangsu nutrition study. PLoS ONE.

[16] Ha J.H., Doguer C., Wang X., Flores S.R., Collins J.F. (2016). High-iron consumption impairs growth and causes copper-deficiency anemia in weanling sprague-dawley rats. PLoS ONE.

[17] Zhang J., Wang C.R., Jin S.H., Song P.K., Meng L.P., Man Q.Q., Jia S.C. (2009). Risk factors on anemia among rural elderly women aged 50–75 y in Xinning county, Anhui province, China. Chin. J. Epidemiol..

[18] Habib M.A., Black K., Soofi S.B., Hussain I., Bhatti Z., Bhutta Z.A., Raynes-Greenow C. (2016). Prevalence and predictors of iron deficiency anemia in children under five years of age in Pakistan, a secondary analysis of national nutrition survey data 2011–2012. PLoS ONE.

[19] Zhai Y., Yin Z.X., Xu J.W., Zeng Y., Liu Y.Z., Shi X.M. (2010). Anemia status and its relevant factors among elderly people aged above 80 years old in longevity areas in China. Chin. J. Prev. Med..

[20] Cespedes E.M., Hu F.B. (2015). Dietary patterns: From nutritional epidemiologic analysis to national guidelines. Am. J. Clin. Nutr..

[21] Ma Y.X., Zhang B., Wang H.J., Du W.W., Su C., Zhang J.G., Zhang J., Zhai F.Y. (2012). Trend on vitamin c intake among Chinese population aged 50–79 years in 9 provinces, from 1991 to 2009. Chin. J. Epidemiol..

[22] Drewnowski A., Rehm C.D. (2014). Consumption of low-calorie sweeteners among U.S. adults is associated with higher healthy eating index (HEI 2005) scores and more physical activity. Nutrients.

[23] Sotos-Prieto M., Luben R., Khaw K.T., Wareham N.J., Forouhi N.G. (2014). The association between Mediterranean Diet Score and glucokinase regulatory protein gene variation on the markers of cardiometabolic risk: An analysis in the European Prospective Investigation into Cancer (EPIC)-Norfolk study. Br. J. Nutr..

[24] He Y., Zhai F., Yang X., Ge K. (2009). The Chinese diet balance index revised. Acta Nutr. Sin..

[25] Wang Y., Li R., Liu D., Dai Z., Liu J., Zhang J., Zhou R., Zeng G. (2016). Evaluation of the dietary quality by diet balance index for pregnancy among pregnant women. J. Hyg. Res..

[26] Cheng K., Li L., Ruan L., Shen Y. (2016). Study on DBI and metabolic syndrome among adults aged 45–60 in Hefei. Food Nutr. China.

[27] Tao X.C., Ge H., Yang L., Wu J. (2015). The association between DBI and bone mineral density in women. Guangdong Med. J..

[28] Yang Y. (2005). Chinese Food Composition Table 2004.

[29] Xu X., Hall J., Byles J., Shi Z. (2015). Assessing dietary quality of older Chinese people using the Chinese diet balance index (DBI). PLoS ONE.

[30] Zang J., Yu H., Zhu Z., Lu Y., Liu C., Yao C., Bai P., Guo C., Jia X., Zou S. (2017). Does the dietary pattern of Shanghai residents change across seasons and area of residence: Assessing dietary quality using the Chinese diet balance index (DBI). Nutrients.

[31] World Health Organization (2011). Haemoglobin Concentrations for the Diagnosis of Anaemia and Assessment of Severity. http://www.who.int/vmnis/indicators/haemoglobin/en/.

[32] Zhou B.F. (2002). Effect of body mass index on all-cause mortality and incidence of cardiovascular diseases—Report for meta-analysis of prospective studies open optimal cut-off points of body mass index in Chinese adults. Biomed. Environ. Sci..

[33] Zhai F.Y., Du S.F., Wang Z.H., Zhang J.G., Du W.W., Popkin B.M. (2014). Dynamics of the Chinese diet and the role of urbanicity, 1991–2011. Obes. Rev..

[34] Wang H.J., Zhang B., Du W.W., Liu A.D., Zhang J.G., Wang Z.H., Su C., Ma Y.X., Zhai F.Y. (2011). Trends of the dietary fiber intake among Chinese aged 18–45 in nine provinces (autonomous region) from 1989 to 2006. Chin. J. Prev Med..

[35] Yang G., Kong L., Zhao W., Wan X., Zhai Y., Chen L.C., Koplan J.P. (2008). Emergence of chronic non-communicable diseases in China. Lancet.

[36] Society C.N. (2008). The Chinese Dietary Guidelines.

[37] Vandenplas Y. (2015). Lactose intolerance. Asia Pac. J. Clin. Nutr..

[38] Wang Y., Li S. (2008). Worldwide trends in dairy production and consumption and calcium intake: Is promoting consumption of dairy products a sustainable solution for inadequate calcium intake?. Food Nutr. Bull..

[39] Liu R., Zhao Y., Yan H., Dang S., Pang S., Wang X., Wang F. (2014). Using the revised Chinese diet balance index Quality of Diet to evaluate the quality of diet among rural residents in Hanzhong, Shaanxi province and relative influencing factors. Chin. J. Epidemiol.

[40] Liu J.P., Cheng J.Q., Peng C.Q., Huang W., Zhang J.Z., Li B., Huang H.X., Pan L.B., Sun Q.L., Luo X.R. (2012). Measuring diet quality of labor workers in Shenzhen using Chinese diet balance index. Chin. J. Prev. Med..

[41] Meng L., Liu J., Zhang J., Wang C., Man Q., Li L. (2009). Effect of dietary factors on anaemia among rural elderly women in south-west China: A case-control study. Pub. Health Nutr..

[42] Lane D.J., Richardson D.R. (2014). The active role of vitamin c in mammalian iron metabolism: Much more than just enhanced iron absorption!. Free Radic. Biol. Med..

[43] Diaz M., Rosado J.L., Allen L.H., Abrams S., Garcia O.P. (2003). The efficacy of a local ascorbic acid-rich food in improving iron absorption from Mexican diets: A field study using stable isotopes. Am. J. Clin. Nutr..

[44] Song P., Li L., Man Q., Wang C., Meng L., Zhang J. (2014). Case-control study of anaemia among middle-aged and elderly women in three rural areas of China. BMJ Open.

[45] Shi Z., Hu X., Yuan B., Pan X., Dai Y., Holmboe-Ottesen G., Byles J.E. (2008). Strong negative association between intake of tofu and anemia among Chinese adults in Jiangsu, China. J. Am. Diet. Assoc..

[46] Shiga K., Hara H., Okano G., Aoyama Y. (2003). Ingestion of water-soluble soybean fiber prevents gastrectomy-induced iron malabsorption, anemia and impairment of voluntary running exercise performance in rats. J. Nutr..

[47] Song P.K., Zhang J., Wang C.R., Li L.X., Man Q.Q., Liu L., Chang F. (2008). Risk factors analysis on anemia among rural women aged 50–75 years in Huangling county, Shanxi, northwest of China. Chin. J. Prev. Med..

[48] Clarys P., Deriemaeker P., Huybrechts I., Hebbelinck M., Mullie P. (2013). Dietary pattern analysis: A comparison between matched vegetarian and omnivorous subjects. Nutr. J..

