# Supplementary material for: Dietary Balance Index-07 and the Risk of Anemia in Middle Aged and Elderly People in Southwest China: A Cross Sectional Study
Source: Nutrients. 2018 Jan 31;10(2):162. doi: 10.3390/nu10020162 (PMC5852738; doi:10.3390/nu10020162)
Supplement: Supplementary file 1 [file nutrients-10-00162-s001.pdf]

Table S1. Components of Chinese dietary balance index revised (DBI-07).

**Components**	**Score**	**Subgroup**	**Score**	**Intake Range by Intake Level (kJ)**						
				**6700**	**7550**	**8350**	**9200**	**10,050**	**10,900**	**11,700**
C1-Cereals*	(−12)–12	Cereals	(−12)–12 **	0~49 = −8<br>200–250 g = 0<br>>500 g = 12	0~25g = −10<br>225~275 g = 0<br>>525 g = 12	<25 g = −12<br>275~325 g = 0<br>>575 g = 12	<25 g = −12<br>275~325 g = 0<br>>575 g = 12	<75 g = −12<br>325~375 g = 0<br>>625 g = 12	−12 < 125 g = −12<br>375~425 g = 0<br>>675 g = 12	<175 g = −12<br>425~475 g = 0<br>>725 g = 12
C2-Vegetables and Fruits	(−12)–0	Vegetables	(−6)–0	≥300 g = 0<br>150~299 g = −2<br>1~149 g = −4<br>0 g = −6		≥350 g = 0<br>175~249 g = −2<br>1~175g = −4<br>0 g = −6	≥400 g = 0<br>200~399 g = −2<br>1~199 g = −4<br>0 g = -6	≥450 g =0<br>225~449 g = −2<br>1~225 g = −4<br>0 g = −6	≥500 g = 0<br>250~499 g = −2<br>1~249 g = −4<br>0 g = −6	
		Fruits	(−6)–0	≥200 g = 0<br>100~199 g = −2<br>1~99 g = −4<br>0 g = −6		≥300 g = 0<br>150~299 g = −2<br>1~149g = −4<br>0 g = −6		≥400 g = 0<br>200~399 g = −2<br>1~199 g = −4<br>0 g = −6		≥500 g = 0<br>250~499 g = −2<br>1~249 g = −4<br>0 g = −6
C3-Milk and dairy products, Soybean and soybean products	(−12)–0	Dairy	(−6)–0	≥ 300 g = 0, score decreased 1 with intake amount decreased 50 g						
		Soybean	(−6)–0	≥30 g = 0, 15~29 g = −2<br>1~14 g = −4, 0 g = −6		≥40 g = 0, 20~39 g = −2<br>1~19 g = −4, 0 g = −6			≥50 g = 0, 25~49 g = −2<br>1~24 g = −4, 0 g = −6	
C4-Animal food	(−12)–(−8)	Red meat, products, Poultry and game	(−4)–4	0 g = −4,<br>1~25 g = −2<br>25~75 g = 0<br>75~125 g = 2<br>>125 g over = 4			0 g = −4<br>1~50 g = −2<br>50~100 g = 0<br>101~150 g =2<br>>150 g = 4			

		Fish,and shrimp	(−4)–0	<20 g = −4<br>20~29 g = −3<br>30~39 g = −2<br>40~49 g = −1<br>≥50 g = 0	<30 g = −4<br>30~44 g = −3<br>45~59 g = −2<br>60~74 g = −1<br>≥75 g = 0	<40 g = −4<br>40~59 g = −3<br>60~79 g = −2<br>80~99 g = −1<br>≥100 g = 0
		Egg	(−4)–4	>75 g = 4, 51~75 g = 2, 25~50 g = 0, 1~24 g = −2, 0 g = −4		
C5-Condiments and alcoholic beverage	0–12	Cooking oil	0–4	≤25 g = 0, 26~50 g = 2, > 50 g = 4		≤30 g = 0, 30~60 g = 2, >60 g = 4
		Salt	0–4	≤6 g = 0, 7~12 g = 2, >12 g = 4		
		Alcohol Beverage	0–4	Male：≤25 g = 0, 26~50 g =1, 51~75g = 2, 76-100g=3, >100g=4<br>(25 g alcohol = 750 mL beer or 250 mL wine or 75 g liquor(<38°)or 50 g liquor(> 38°)<br>Female：≤15 g = 0, 15~30 g = 1, 31~45 g = 2, 46~60 g = 3, >60 g = 4<br>(15 g alcohol = 450 mL beer or 150 mL wine or 50 g liquor(38°) or 30 g liquor(> 38°)		
C6-Diet variety	(−12)–0	Diet variety	(−12)–0	for consumption of greater than 25 g of foods (soybean is 5 g), otherwise score is −1		
C7-Drinking water	(−12)–0	Drinking water	(−12)–0	> 1200 mL = 0, <100 mL = −12<br>score decreased 1 with intake amount decreased 100 mL from 0 to 12		

* Cereal include rice, wheat，dried legumes(exclude soybean) and tubers. Intake amount means fresh amount. Sweat potato：intake amount divided by 3；
potato：intake amount divided by 4；yam and yambean：divided by 6；** score increased (decreased) 2 with 50 g intake increased (decreased) from 0 to maximal(minimal) score

Table S2. Elements of diet varety score

Food subgroups	Score	Representative foods
F1-Rice and products	-1,0	Steamed/boiled rice, rice products
F2-Wheat and products	-1,0	Wheat bun, wheat noodles, wheat pancake
F3-Corn, coarse grains and products, Starchy roots and products	-1,0	Corn, barley grain, foxtail millet, buckwheat, sweet potato, yam, taro, potato, green bean, read bean
F4-Dark-colored vegtables	-1,0	Spinach, carrot, tomato
F5-Light- colored vegtables	-1,0	Cabbage, cucumber, pickles
F6-Fruits	-1,0	Fresh and dried fruits
F7-Soybean and products	-1,0	Soybean, black bean, bean curd
F8-Milk and dairy products	-1,0	Milk, milk powder, cheese
F9-Red meat and products	-1,0	Beef, pork, lamb, liver, sausage
F10-Poultry and game	-1,0	Chicken, duck, rabbit
F11-egg	-1,0	Hen egg, duck egg
F12-Fish and selfish	-1,0	Fish, shrimp, mussel

Table S3. The Dietary Guidelines for Chinese residence and the corresponding components for Diet Balance Index revised (DBI-07)

	**Dietary guidelines for Chinese residents**	**Corresponding component(s) in the DBI-07 components**
1	Eat a variety of foods, with cereals as the staple and include a certain amount of coarse grains	Diet variety , cereals
2	Consume plenty of vegetables, fruit and tubers	Vegetables and fruit
3	Consume milk, soybean, or dairy- or their products every day	Dairy , soybean
4	Consume appropriate amounts of fish, poultry, eggs and lean meat;	Animal food
5	Reduce cooking oil, choose a light diet that is also low in salt.	Cooking oil , salt
6	Avoid over eating, exercise every day, to maintain a healthy body weight.	
7	Rationally distribute the daily food intake among the three meals, if you take snacks, do so appropriately	
8	Drink sufficient water every day, rationally choose beverages	Drinking water
9	If you drink alcoholic beverages, do so in limited amounts	Alcoholic beverages
10	Avoid unsanitary and spoiled foods	
